# Hypothesis: Spontaneous Advent of the Prebiotic Translation System via the Accumulation of L-Shaped RNA Elements

**DOI:** 10.3390/ijms19124021

**Published:** 2018-12-12

**Authors:** Ilana Agmon

**Affiliations:** 1 Institute for Advanced Studies in Theoretical Chemistry, Schulich Faculty of Chemistry-Technion-Israel Institute of Technology, Haifa 3200003, Israel; chilana@technion.ac.il; Tel.: +972-4-8293824; 2 Fritz Haber Research Center for Molecular Dynamics, Hebrew University, Jerusalem, 9190401, Israel

**Keywords:** evolution, origin of life, ribosome, proto-ribosome, tRNA, translation

## Abstract

The feasibility of self-assembly of a translation system from prebiotic random RNA chains is a question that is central to the ability to conceive life emerging by natural processes. The spontaneous materialization of a translation system would have required the autonomous formation of proto-transfer RNA (tRNA) and proto-ribosome molecules that are indispensable for translating an RNA chain into a polypeptide. Currently, the vestiges of a non-coded proto-ribosome, which could have only catalyzed the formation of a peptide bond between random amino acids, is consensually localized in the region encircling the peptidyl transferase center of the ribosomal large subunit. The work presented here suggests, based on high resolution structures of ribosomes complexed with messenger RNA (mRNA) and tRNAs, that three types of L-shaped RNA building blocks derived from the modern ribosome, alongside with an L-shaped proto-tRNA, each composed of about 70-mer, could have randomly occurred in the prebiotic world and combined to form a simple translation system. The model of the initial coded proto-ribosome, which includes the active sites of both ribosomal subunits, together with a bridging element, incorporates less than 6% of the current prokaryotic rRNA, yet it integrates all of the ribosomal components that are vital for synthesizing the earliest coded polypeptides.

## 1. Introduction

The modern translation system is a complex assembly of biomolecules that co-operate to produce proteins, the principal constituents of every organism. Synthesis of polypeptides, which in turn can fold into proteins, takes place at the ribosome and is directed by the instructions that are held in a copy of the gene coding for the protein sequence, i.e., by the messenger RNA (mRNA). The ribosome is an enormous complex of RNA and proteins, composed of two subunits, the large subunit (LSU) and the small subunit (SSU), having a total molecular weight of approximately 2.5 MDa in bacteria. About two-thirds of the bacterial ribosome mass consists of RNA, inclusive of the active sites of both subunits, classifying the ribosome as a ribozyme. Proteins, which constitute the remaining third of the mass, serve mostly in auxiliary roles. The active site of the large subunit, the peptidyl transferase center (PTC), is the location in which new amino acids are incorporated into the growing peptide chain, while the small subunit accommodates the mRNA and contains the decoding center.

The genetic code is applied via the mediation of transfer RNA (tRNA) molecules, which link the mRNA codons with their matching amino acids. The modern tRNA is an L-shaped molecule. One end of the molecule is the universally conserved single stranded NCCA tail, which carries the cognate amino acid (aa), while the anticodon (AC) loop is located at the other end. Prior to peptide bond formation, two tRNA molecules, the aminoacyl tRNA (A-tRNA) carrying the incoming amino acid, and the peptidyl-tRNA (P-tRNA) carrying the peptidyl chain synthesized up to that stage, are accommodated at the A- and P-sites of both subunits. The AC loops of the A- and P-tRNAs are base-paired to adjacent codons on mRNA, while their 3′ ends position the reacting amino acids in the PTC, in a stereochemistry that is suitable for peptide bond formation, resulting in the lengthening of the nascent chain by one amino acid at each elongation cycle.

The advent of the elaborate contemporary translation system by mere chance is highly unlikely. A prebiotic system possessing the capability to translate RNA chains into peptides could have emerged spontaneously only if it was far less complex. The core elements of the modern translation system, i.e., the catalytic regions of the ribosome and the tRNAs, consist of RNA. Evolutionary continuity entails that models of the prebiotic translation system should thus be composed of RNA elements as well. RNA possesses idiosyncratic characteristics: 1.) Oligonucleotides tend to fold spontaneously into stable 3D structures, a feature that can grant them with catalytic capabilities. 2.) RNA helices possess intrinsic flexibility, allowing the formation of kinks that can be used to store energy in biological motors. 3.) The replication mode of RNA, via nucleotide complementarity, may enable reliable copying, even under primitive conditions. Taken together, these features could have promoted the formation and sustainability of a prebiotic translation system.

Various hypotheses, aimed at presenting simpler versions of the components of the modern translation system and of its mechanism, were put forward. The contemporary tRNA molecules were proposed to be preceded by hairpins [1,2,3,4,5,6], and a process by which two charged hairpins, lined up side by side along an RNA string serving as mRNA, was suggested to have provided the earliest mechanism for the formation of peptides [6,7,8,9]. A decoding region analog composed of a 49-mer RNA helix, which contains the decoding center as derived from h44 of the SSU, capped with a tetraloop at one end and initiated by a six base-pairs helix at the other end, was synthesized. It was shown to interact with antibiotics, and with RNA ligands specific to the SSU, in a manner that is analogous to that of the contemporary small subunit, suggesting that the complexity of the modern ribosome can be circumvented to some degree [10].

**Figure 1:**
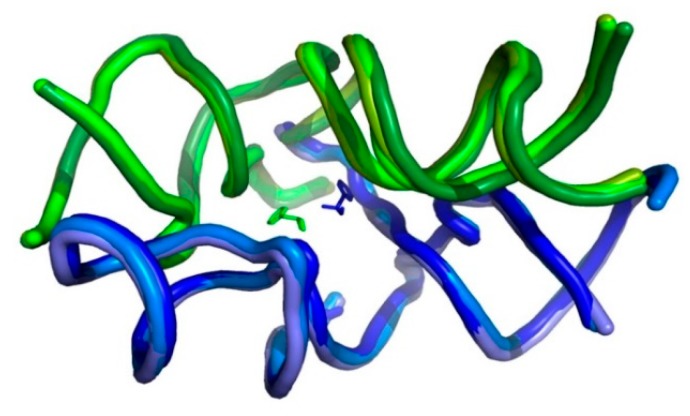
The Dimeric Proto-Ribosome (DPR) pocket. Overlap of the DPR fold, with the Peptidyl Transferase Center (PTC) at its core, as found in the high resolution structures of archaea (Protein Data Bank (PDB) code 1VQ6), bacteria (PDB code 2WDL), and eukarya (PDB code 3U5D), portraying the extreme tertiary conservation of this region. A-DPR monomers in blue hues, P-DPR monomers in green hues, with the reactants (PDB code 2WDL) positioned at the bottom of the cavity. The pocket is projected approximately along the symmetry axis. The elbows of the L-shape monomers point into the page.

Hypotheses putting forward simplified versions of the contemporary ribosome have consensually located its vestige in the region encircling the current PTC [11,12,13,14,15,16,17]. Models derived from the modern ribosome offered pocket-like structures that could have accommodated two random amino acids, analogous to the manner by which the current reactants sit within the PTC. This pocket-like structure would have provided positional catalysis, producing short peptides with random composition. The smallest and simplest contender for being a non-coded proto-ribosome of dimeric nature is the DPR (dimeric proto-ribosome) [15,18], whose monomers, being part of the symmetrical region [19] (Figure S1), are similar to each other in fold and in nucleotide conformation. The L-shaped monomers are positioned around an approximate 2-fold rotation axis, together assembling the PTC pocket (Figure 1). The DPR model offers several advantages: 1.) its pocket conserves the PTC layout, enabling the mutual positioning of the reactants in favorable stereochemistry 2.) Sequences prone to fold and dimerize spontaneously to form a DPR-like pocket were shown to have a realistic probability to occur randomly in the prebiotic world [18]. 3.) The sequence complementarity between nucleotides that constitute the two halves of the PTC cavity, observed in bacterial ribosomes, indicates an efficient replication mode for a dimeric proto-ribosome [20].

The work presented here uses high-resolution X-ray structures of bacterial ribosomes complexed with mRNA and three tRNA molecules, to extract basic components that could have assembled the prebiotic coded translation system. Geometrical restrictions are shown to imply that the simplest tRNA that could have been involved in the prebiotic translation would be an L-shape molecule. This molecule, together with three different types of building blocks derived from the modern ribosome, all being L-shape structures composed of RNA sequences of about 70-mer, are suggested to have provided the necessary components for translating RNA chains into polypeptides in the prebiotic world. 

## 2. Results and Discussion

### 2.1. Shape of the Ancestral tRNA

**Figure 2:**
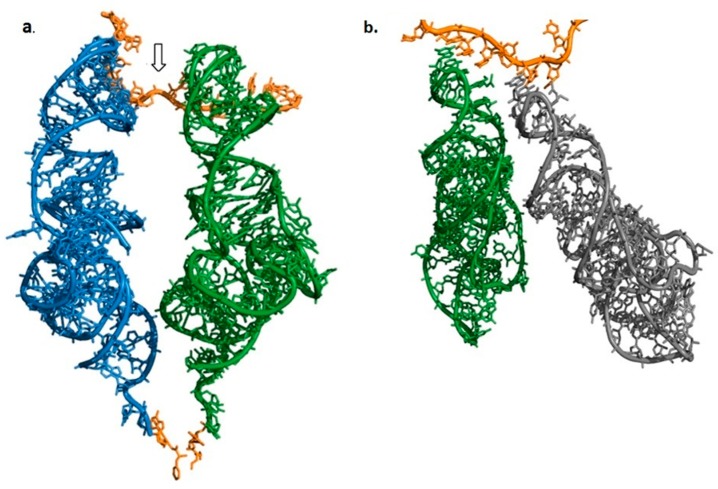
Juxtaposition of two tRNA molecules attached to adjacent codons on the mRNA. A-site tRNA in dark blue, P-site tRNA in dark green, E-site tRNA in gray, mRNA in orange, and the ultimate residue A76 with the attached amino acid in gold. (**a**) A-, P-site tRNAs (PDB code 1VY4) forming a rhombus-like arrangement. The kink in mRNA is marked by an arrow. (**b**) P-, E-site tRNAs, which are paired to a less kinked segment of mRNA (PDB code 4V6F), deflect.

The minimum prerequisite from aminoacylated tRNAs involved in the prebiotic translation, would be their ability to line up side by side, being base-paired to an oligonucleotide serving as mRNA, while positioning the reacting amino acids in a proximity that allows for the formation of a coded peptide bond. The stereochemistry of a complex of two charged tRNAs attached to an mRNA was studied, using the high resolution structures of the modern 70S bacterial ribosomes (Materials and Methods). The length of a nucleotide-triplet, as obtained from measurements carried out on the mRNAs, lay in the range 13.8–18.2 Å, depending on the curvature of each codon, while the range of the maximal width of the double stranded AC stem, as obtained from measurements carried out on the tRNAs, is 20.6–21.7 Å (Figure S2). Parallel accommodation of two tRNAs side by side, would have thus led to a severe clash between their AC stems. To circumvent this steric constraint, the reading of the two adjacent codons in the modern ribosome is accomplished by a significant kink of about 105° in the mRNA backbone between the A- and P-site codons, which results in an angle of about 26° between the planes of the A- and P-tRNAs [21] (Figure 2a), forming a rhombus-like configuration that prevents the collision. In order to bring the two amino acids attached to the 3′ends of the A-, P-tRNAs into the required proximity, the acceptor-TΨC arms of the modern A-, P-tRNAs approach each other in an inclined manner, up to a distance of 15.8±1.2 Å between the O3′ atoms of nucleotide A72 in A- and P-tRNAs (Figure S2). The proximity allows the NCCA tails attached to these atoms to position the reacting amino acids in the PTC with the stereochemistry required for peptide bond formation. In the case of a moderate kink of 138° in the mRNA backbone, the juxtaposition of two tRNAs, as demonstrated by the AC stems of the P-, E-site tRNAs (PDB code 4V6F), leads to the deflection of the stems (Figure 2b). When the mRNA backbone between the P- and E-sites is nearly straight (PDB code 1VY4), the E-site tRNA is not base paired to the mRNA at all. 

Replacing the A-, P-site tRNAs by hairpins, in order to test the hypothesis that two hairpins attached to adjacent codons on mRNA could have induced the prebiotic polypeptide synthesis [6,7,8,9], alters the stereochemistry of the reactants completely. The accommodation of two hairpins of four base-pairs on an mRNA, for example, will double the distance between the corresponding positions to which the 3′ end tails would be attached. The distance between O3′ of nucleotide 42 in the A- and P-tRNAs accommodated on the ribosome is about 37.1±0.3 Å (Figure S2), and it would be further augmented for longer hairpins, precluding any proximity between their 3′ ends.

These observations, which are independent of any element that is specific to the contemporary translation system, indicate that hairpins would have failed to take part in the primitive translation. Avoiding the collision of the stems by a kink in the translated RNA or by deflection of the stems (Figure 2a,b), would have set apart the reactants that are attached to the hairpins, obstructing any chemistry involving the aa-3′ ends. Moreover, the proximity between the 3′ends of the acceptor stems, which is made possible by the incline of the acceptor-TΨC stems towards each other, would be an impossible configuration for two parallel hairpins paired to neighboring codons. The stereoscopic difficulty was noticed by Orgel, even though the structural data available at the time was incomplete; “A striking feature of protein synthesis is the use of contiguous base triplets in the message, to code for contiguous amino acid. Our model provides no obvious reason why this should be true considering the crowding that would be expected at the junction between a message and 2 pre-tRNAs”. Correspondingly, he proposed a rather complex structure for the prebiotic tRNA that allowed the acceptor stems to slant towards one another [22].

**Figure 3:**
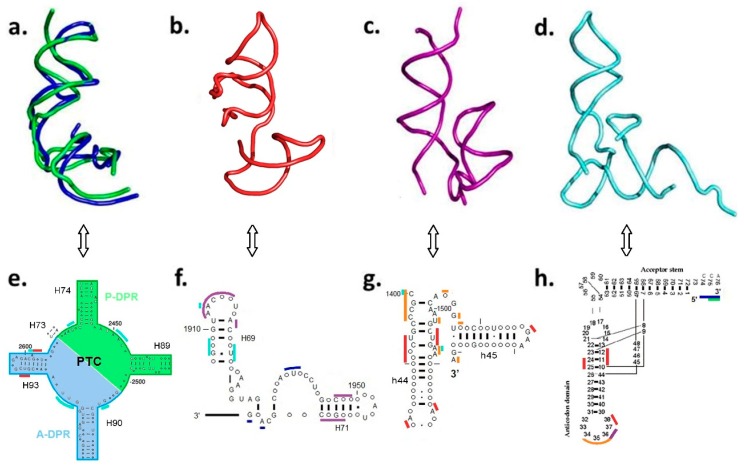
Building blocks of the initial translation system model. (**a**) Overlap of the symmetry-related A- and P-DPR elements assembling the non-coded proto-ribosome (**b**) the H69–71 bridging element, (**c**) the h44–45 proto-SSU, (**d**) tRNA, (**e**–**h**) the secondary schemes of the four building blocks. Highly conserved nucleotides (materials and methods) are presented by capital letters and nucleotides involved in interactions among the different types of building-block are marked by a contour line having the color of the interacting block: A-DPR (blue), P-DPR (green), H69–71 bridging element (red), h44–45 proto-SSU (purple), tRNAs (cyan), mRNA (orange). The same color code is maintained in Figure 3, Figure 4, Figure 5 and Figure 6.

These geometrical restrictions, combined with evolutionary continuity, entail that the proto-tRNAs involved in the prebiotic translation process would have had an L-shape conformation. However, the intricate elbow region of the modern tRNA, having an angle of about 90° adjusted to the current stereochemistry of the ribosome, could have possibly evolved later. An elemental shape made up of two helices connected by a simpler elbow region, possibly similar to the elbow region of the DPR monomer encapsulating the PTC (Figure 3a), would suffice for accomplishing the simultaneous interactions of the proto-tRNA with the ancient mRNA and with the PTC, which constitutes an indispensable prerequisite for translating a code into a peptide.

### 2.2. A Model of a Coded Proto-Ribosome 

The universal nature of the contemporary ribosome, which employs a common mode of action in all life domains, together with its considerable level of structural and phylogenetic conservation, implies that the essence of the current translation mechanism should have already been present in the Last Universal Common Ancestor (LUCA). It follows that the vestige of the stand-alone primordial proto-ribosome, which is assumed to have continuously evolved into the modern ribosome, may still be inferred from the contemporary structure. A model of a coded proto-ribosome, i.e., an entity possessing the capability to translate a code embodied in an RNA template into a polypeptide, should therefore include the two active sites; an RNA element that accommodates mRNA and contains the decoding center, which is derived from the ribosomal RNA (rRNA) of the SSU i.e., a proto-SSU, and an element that forms the PTC pocket, derived from the rRNA of the LSU. The spatial gap between these elements necessitates the existence of a bridging element that guarantees the structural continuity of the model. Adherence to the current translation mechanism entails that the model should also retain the stereochemistry of the interactions of the two L-shape tRNA molecules with the active sites of both subunits. The minimal model for an initial system involved in a processive translation of RNA chains into polypeptides, will thus include three types of building blocks derived from the rRNA of the modern ribosome (Figure 3a–c), two L-shape tRNAs (Figure 3d), RNA chains acting as mRNA and amino acids.

The prebiotic PTC pocket will be approximated by the DPR (Figure 1) derived from the LSU structure, due to the DPR’s compatibility with the fundamental expectation from a self-materializing entity, i.e., the realistic probability to emerge autonomously while retaining the PTC layout [15,18]. The DPR is composed of two symmetrically positioned monomers, the A-, P-DPR, each being an L-shape RNA element (Figure 3a,e), which, within the modern ribosome, are comprised of 60- and 61-mer sequences, respectively. The PTC walls are built chiefly from nucleotides belonging to the central loop of domain V, while the four helices that symmetrically radiate from it, i.e., H74, H89, H90, and H93, stabilize the pocket [19]. Three of these helices, H74, H89, and H90, lack capping within the ribosome (Figures 3e and S1a,b). Completing them with tetraloops to achieve sequence continuity, would have resulted in 64- and 69-mer stand-alone monomers. 

The ancient tRNA is assumed, according to the results of Section 2.1, to have an L-shape conformation as well. It is possible that its sequence was shorter than the modern canonical 76 nucleotides, and that its elbow region was simpler, but for the present purpose, the ancient tRNA will be approximated by the modern one. 

**Figure 4:**
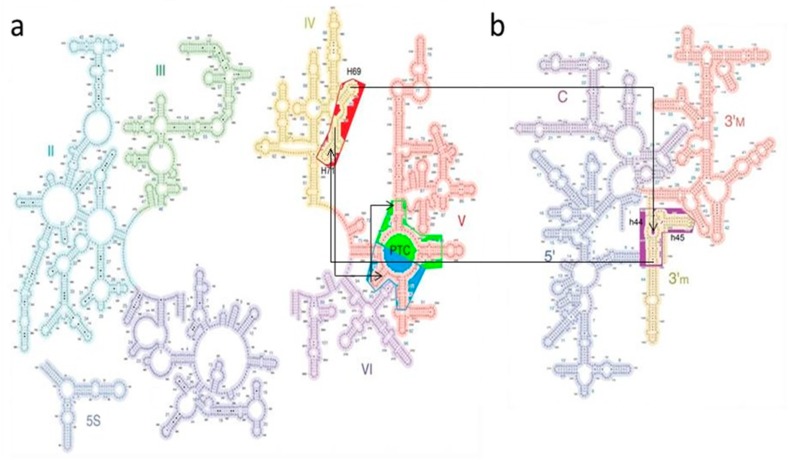
Building blocks of the suggested coded proto-ribosome, depicted on a secondary scheme of the LSU (**a**) and SSU (**b**). The symmetry-related A-DPR (blue background) and P-DPR (green background) monomers, together with the H69–71 bridging element (dark red background), form the suggested proto-LSU, and h44–45 (purple background) forms the proto-SSU. Nucleotides and base-pairs involved in A-minor interactions are connected by arrows.

While the dimensions of the DPR and of the tRNA components are postulated, the extent of the two novel proto-ribosome building blocks, i.e., the proto-SSU that includes h44, h45, and the bridging element that includes H69–H71, were determined via their mutual interactions with the other elements. All of the nucleotides from these regions, which are within an interaction distance of 3.5 Å to the atoms in the other RNA elements composing the translation system model, were incorporated (Table S1–S4). This resulted in a proto-SSU building block composed of nucleotides 1400–1419 and 1481–1504 from h44 (*E. coli* numbering throughout), and 1505–1531 from h45 (Figure 3c,g), forming a 71-mer RNA element. The bridging element contains nucleotides from domain IV, i.e., H69, H71, and the loop connecting them, a loop whose level of phylogenetic conservation nearly reaches that of the highly conserved central loop of domain V that assembles the PTC. Interactions with A-DPR, h44–45 proto-SSU, and with the A-, P-tRNA confine the bridging element to nucleotides 1906–1968, forming a rather complex 63-mer L-shape RNA element (Figure 3b,f). The combination of the DPR with the H69–71 bridging element is suggested to constitute the entire proto-LSU, which together with the proto-SSU holds 267 nucleotides, i.e., less than 6% of the contemporary rRNA (Figure 4).

**Figure 5:**
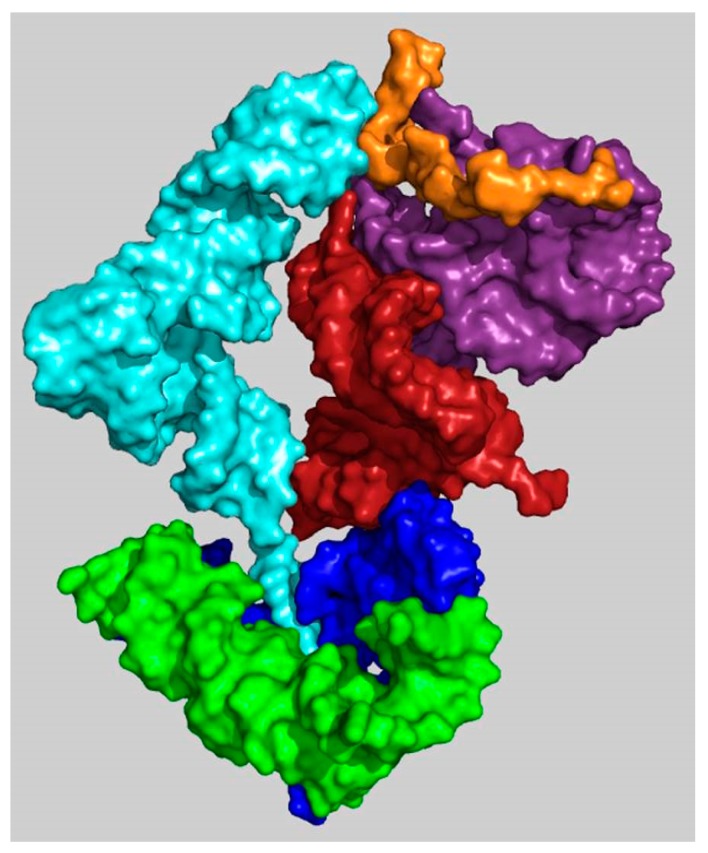
A model of the initial translation system. Three types of ribosomal building blocks forming a simple coded proto-ribosome: 1. A-, P-DPR monomers (blue and green, respectively) 2. H69–71 bridging element (dark red) 3. the h44–45 proto-SSU (purple), which are complemented by tRNA molecules (cyan) interacting with mRNA (orange) and with the PTC pocket (PDB code 1VY4). For clarity, only A-site tRNA is shown.

The three types of L-shape building blocks derived from the rRNA of the contemporary ribosome, are interconnected (Figure 3e–g, Tables S1–S4). P-DPR is connected solely to A-DPR, A-DPR to the H69–71 bridging element, and the bridging element to h44–45 proto-SSU. These constituents together form the model of a coded proto-ribosome (Figure 5). The linking among the building blocks is achieved via A-minor interactions [23] that hook each building block to the neighboring ones (Table 1, Figure 4), as well as by a network of hydrogen bonds and stacking interactions. 

This coded proto-ribosome would be stabilized by the interactions of the tRNA AC-loop with the mRNA accommodated on the h44–45 proto-SSU, and by the positioning of the aa-3′ends in the PTC pocket (Figure 5). Additionally, a wide contact surface formed between the P-tRNA and the H69–71 bridging element (Table S4) would have provided further stabilization, giving rise to the assembly of a compact system that contains all the RNA components crucial for translating a code prebiotically. 

### 2.3. Spontaneous Formation of the Coded Proto-Ribosome Components

The fold of a hypothetical stand-alone RNA element, which is assumed to have materialized spontaneously in the prebiotic world, can be predicted by applying free energy minimization to its sequence. Predictions of the secondary schemes of the A- and P-DPR monomers, using sequences derived from structures of contemporary prokaryotic ribosomes, demonstrated that if any of these sequences existed in the prebiotic world, it would have been compelled to fold into an L-shape element matching the one found within the ribosome. Moreover, RNA sequences of about 70-mer, having the tendency to fold into DPR-monomer-like structures that conserve the reactant’s accommodation position in the PTC and dimerize in an energetic down-hill process, were shown to have a realistic probability to occur randomly [15,18]. The DPR pocket, found in the core of the modern ribosome, could have therefore been assembled spontaneously in the prebiotic world. 

Fold predictions of the proto-SSU and of the bridging element, using their *E. coli* and *Thermus thermophilus* sequences, were performed by Mfold [26] (materials and methods), a program that screens the fold-landscape of an input sequence and produces a list of thermodynamically favorable secondary structures, along with their free energy of formation. The sequence of H69–71 bridging element, which is common to both bacteria, acquired a fold analogous to that which is found within the current ribosome (Figure S3a,b). The sequences of the proto-SSU element, which differ slightly in *E. coli* and *Thermus thermophilus*, fold into an identical structure (Figure S3c,d). The fold predicted for the h45 sequence is the same as the one found within the ribosome, but the base-pairing pattern of the section of h44 contained in the proto-SSU is rearranged, possibly because this region had to undergo massive mutational variation in order to fulfil its intricate contemporary role. The folding free energy of the bridging element was ΔG = −19.1 kcal/mol, and that of the proto-SSU ΔG = −34.4 ± 2 kcal/mol, indicating the self-foldability and stability of the obtained structures.

### 2.4. Evolutionary Path of the Translation System

A mandatory prerequisite for the spontaneous advent of the building blocks assembling the model of the initial translation system (Figure 5), would be the prebiotic existence of random RNA chains of about 70-mer. Long oligonucleotides, reaching over 100-mer, were demonstrated, both experimentally and theoretically, to polymerize in solution under a thermal gradient [27]. Long oligonucleotides, obtained in this manner or otherwise, could have autonomously folded into stable RNA molecules, mostly hairpins and RNA elements with two helical arms [28]. Various types of L-shape RNA molecules could have therefore been floating around in the prebiotic environment, being self-folded products of the prebiotic chemistry. Among these, L-shape RNA elements resembling the DPR monomers, the H69–71 bridging unit, the h44–45 proto-SSU and the tRNAs could have been present. The sequences of the first three building blocks can be extracted from the conserved modern ribosomes, permitting the prediction of their L-shape secondary structures. The proto-tRNA molecules, whose sequences cannot be fully retraced, were suggested to have acquired an L-shape conformation via the association of two matching hairpins [29,30,31], or due to the stability granted by this type of fold [32]. During the review process, it was brought to my attention that a competing model for tRNA evolution, via the ligation of three minihelices, has been proposed [33,34].

**Figure 6:**
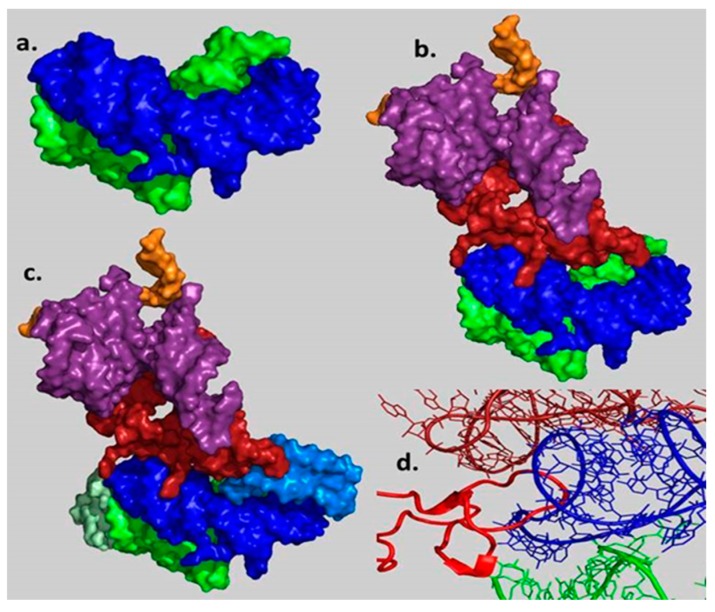
Evolution of the proto-ribosome (**a**) Stage 1: side view of the non-coding DPR pocket suggested to catalyze the peptide bond formation between random amino acids. (**b**) Stage 2: rear view (rotated by 90° around the vertical axis relative to Figure 5) of the initial coded proto-ribosome model capable of processively translating an RNA chain into a polypeptide. (**c**) Stage 3: the same view as in (**b**) after the addition of the A- and P-sites (in lighter hues) to the DPR, resulting in a higher connectivity with the bridging element. (**d**) Stage 4: protein incorporation. Interactions of the L2 loop (in red), suggested to be the first protein to join the coded proto-ribosome, with the A-, P-DPR and with the tail of the bridging element.

In the first stage of the ribosome evolution (Figure 1 and Figure 6a) two DPR-monomers, similar in fold and nucleotide conformation but differing in sequence, could have coincidentally bumped into each other and dimerized, forming the PTC pocket. This step, which occurs in an energetically down-hill process, would have yielded the DPR, an early non-coded proto-ribosome that could have catalyzed the association of arbitrary amino acids, producing short peptides of random sequences [15,18].

In the second stage (Figure 6b) the two other building blocks, i.e., H69–71 bridging element and h44–45 proto-SSU, would have joined the DPR, primarily by hooking via A-minor interactions. This mode of assembly is consistent with the hypothesis that A-minor interactions were used by evolution to add new RNA elements to the evolving proto-ribosome [14]. The modern translation process is initiated by base pairing of the initiator tRNA to the start codon on mRNA, followed by the association of the small and the large subunits to form a working ribosome. It is conceivable that the ancient initiation step could have been analogous, i.e., that base-pairing of an aa-proto-tRNA to a codon of an RNA oligonucleotide accommodated on the proto-SSU element, followed by the association with the proto-LSU, would have given rise to the first coded proto-ribosome apparatus. This RNA complex, in cooperation with aminoacylated tRNA molecules, would have constituted a prebiotic system comprising the essential components for translating a code into a polypeptide.

The next evolutionary stage (Figure 6c) is likely to be concerned with the addition of the H92 and H80, stemming from the far ends of H90 and H74, respectively (Figure S1), to the DPR, together assembling the entire symmetrical region [19]. This expansion would have added stabilization to the initial model, due to the large binding interface formed between H92 of the expanded PTC region, and H71 from the bridging element (Table S5). Moreover, the expansion would have stabilized the accommodation of the reactants in the PTC, allowing base pairing between C74, C75 from the tRNA 3′end, and the H92, H80 stem loops, i.e., the A-, P-loops.

The first protein to have been intercalated into the all-RNA coded proto-ribosome, according to the present model, would be L2. This universal r-protein was previously assumed to be among the most ancient ones [35]. Its suggested primacy, in the context of the present study, stems from its being the only contemporary protein that interacts with more than a single building block of the coded proto-ribosome model. While as many as seven proteins interact with a single constituent of the model, L2 interacts with A-DPR, P-DPR, and with U1971 (Table S6), a nucleotide located at the extension of the H69–71 bridging element tail (Figure 6d). In *Thermus thermophilus*, the L2 residues involved in the interactions with the coded proto-ribosome components are 228–243, which closely overlap the initial phase suggested for L2, i.e., residues 226–242 [36]. This 16-mer peptide could have thus provided structural stabilization at the very early stage of ribosome evolution, and could have possibly been involved in the dynamics of the PTC [19]. A translation system in which this peptide was synthesized by chance from a random RNA chain encoding for its sequence, would have therefore been granted with an evolutionary advantage, enhancing its translation fidelity and promoting its proliferation [37].

Assembling a working translation system out of merely four types of L-shape building blocks, random RNA chains and amino acids, excluding any other enzymatic agents, seems to require the existence of two additional processes. An energy-free translocation process may be needed, due to the absence of a GTP center in the building blocks composing the coded proto-ribosome model, and a non-enzymatic process for aminoacylating tRNAs is required, due to the absence of a synthetase. Two experimental results reported in the literature may perhaps be pertinent. The existence of an energy-free translocation process may be implied by the polyU-directed elongation obtained in the absence of EF-Tu, EF-G, and GTP [38], while charging tRNA with its cognate amino acid in the absence of a synthetase or ATP was reported to occur spontaneously under high pressure [39]. However, the relevance of such non-enzymatic processes to prebiotic translation remains to be substantiated, and the absence of such processes would require the addition of enzymatic agents to the proposed initial translation system.

Sustainability of the initial translation system would have required the replication of its RNA components. An appealing non-enzymatic process, assumed to have preceded the enzymatic copying, is offered by the chemical copying of polynucleotides, where activated helper oligonucleotides were shown to catalyze the replication of an RNA template [40]. Interestingly, copying the DPR monomers would have been particularly efficient, due to the sequence complementarity between nucleotides that constitute the two halves of the PTC cavity, observed in bacterial ribosomes. This complementarity implies that the sequence of each monomer could have acted as a template for the synthesis of its counterpart, eliminating the need for two replication steps for each copying event [20]. Variations introduced during the primitive replication phase would have launched Darwinian evolution at a molecular level, promoting selection for the fittest proto-ribosomes. More efficient proto-ribosomes could have, in turn, translated longer random RNA chains, maybe yielding this way minimal versions of the polymerase and of the synthetase. The random RNA chains coding for these primitive enzymes would have been present in situ, allowing their re-translation and enabling a co-evolution process involving the extant ribozymes and enzymes [41], opening the way to life as we know it. The model presented here put forward a continuous evolutionary path, from random RNA chains to life as we know it, initiated via a coded proto-ribosome that seems sufficiently simple to materialize spontaneously in the prebiotic world and perform programmed translation, yielding the first coded polypeptides. 

## 3. Materials and Methods 

The building blocks of the initial translation system model, were derived from the X-ray structures of the bacterial 70S ribosome determined at resolution higher than 3.1 Å, which contain cognate A- and P-tRNAs in a canonical conformation, paired to an mRNA, and an E-tRNA at the exit site. To avoid structural changes caused by entities that could not have existed in the prebiotic environment, structures that include factors, antibiotics, and unusual codons were excluded. Measurements were performed on two 70S ribosomes from *Thermus thermophilus* (PDB codes 1VY4, 4V6F), each containing two molecules in the asymmetric unit. The atomic distances pertaining to the ancient tRNA model were obtained with COOT [42], by averaging the values of the four measurements from the two structures. The distance (d) is given along with the Δ value, d±ΔÅ, where Δ marks the discrepancy between the most remote measurement and the average. The length-range of an mRNA codon was obtained from the 70 triplets found in the four mRNAs examined.

The conservation level of individual nucleotides was obtained from the CRW site [43]. Nucleotides having the same identity in the three life domains, with a conservation level of above 97% in each of the domains, are assumed to be highly conserved.

The secondary structures of RNA oligonucleotides were predicted by applying free energy minimization using the program Mfold [26] version 3.5, with default parameters, and a folding temperature fixed at 37 °C.

## 4. Conclusion

The feasibility of the spontaneous emergence of life via natural processes is highly controversial. A central claim against the scientific approach emphasizes the improbability that the initial stages of emergence could have occurred autonomously. Here, the spontaneous folding of RNA sequences derived from the structure of the modern ribosome, into stable L-shape entities that can assemble to form a compact proto-ribosome, is demonstrated. This autonomously-materializing prebiotic entity, that contains all the RNA components crucial for translating a code into a polypeptide, presents a feasible starting point, obtained via natural processes, for a continuous evolutionary path leading from the prebiotic era to life as we know it. 

## Figures and Tables

**Table 1:** A-minor interactions between the building blocks of the coded proto-ribosomal model.

A-Minor Interaction	Nucleotides Involved (Location)	Contemporary Role
A-DPR → P-DPR	A2598 → C2073:G2436 (H93 stem loop) (H74 stem)	Part of the GNRA interaction between A-, P-DPR [15] (Figure S1)
H69–71 bridging element → A-DPR [14]	A1966 → G2592:C2601 (H71–H67 loop) (H93 stem)	
H69–71 bridging element → h44–45 proto-SSU [24]	A1912 → C1407:G1494 (H69 stem loop) (h44 stem)	Part of the B2a intersubunit bridge [24]
h44–45 proto-SSU → H69–71 bridging element [24]	a. A1418 → G1948:C1958 (h44 stem) (H71 stem) b. A1483 → C1947:G1959 (h44 stem) (H71 stem)	Part of the B3 intersubunit bridge [24] and center of the intersubunit ratchet [25]

## Supplementary Materials

Supplementary materials can be found at http://www.mdpi.com/1422-0067/19/12/4021/s1.

## Abbreviations

DPR	Dimeric Proto Ribosome
aa	Amino Acid
PTC	Peptidyl Transferase Center
LSU	Large Subunit of the Ribosome
SSU	Small Subunit of the Ribosome
AC	Anticodon
rRNA	Ribosomal RNA
A-tRNA	A-site tRNA
P-tRNA	P-site tRNA
