# Supplementary material for: Hypothesis: Spontaneous Advent of the Prebiotic Translation System via the Accumulation of L-Shaped RNA Elements
Source: Int J Mol Sci. 2018 Dec 12;19(12):4021. doi: 10.3390/ijms19124021 (PMC6321417; doi:10.3390/ijms19124021)
Supplement: Supplementary file 1 [file ijms-19-04021-s001.pdf]

Supplementary Tables S1–S6 – List of interaction distances shorter than 3.5 Å, found between residues composing the components of the initial translation system model. Distances were calculated from atomic coordinates of the bacterial ribosome structure from *Thermus thermophilus* (PDB code 1VY4).
*- nucleotides listed under this symbol belong to the component tagged with *.
#- nucleotides listed under this symbol belong to the component tagged with #.

Table S1. Interactions of the A-, P-DPR nucleotides.

Component	P-DPR *
A-DPR #	* ~ #
	A2059 ~ A2503
	A2060 ~ G2502
	G2061-2 ~ A2503
	C2073 ~ A2598
	U2074 ~ G2597, A2598
	G2436-8 ~ A2598 - A2600
	A2439 ~ C2586, A2587, A2600
	C2441 ~ C2586
	G2447 ~ U2504
	C2452 ~ U2504
	A2453 ~ U2504, A2572
	U2491 ~ G2569, G2570
	U2500 ~ U2504
	C2501 ~ G2502

Table S2. Interactions between A-DPR, H69-71 bridging element and h44-45 proto-SSU.

Component	A-DPR *	H69-71 bridging element *
H69-71 bridging element #	* ~ #	x
	C2591 ~ A1939	
	G2592 ~ A1939, A1966, G1968	
	U2593 ~ A1966	
	G2603 ~ U1940	
	U2604 ~ U1939	
	U2605 ~ A1938	
h44-45 proto-SSU #	NO INTERACTION	* ~ #
		A1912 ~ C1407, A1408, G1494
		A1913 ~ G1494
		C1914-5 ~ C1409
		A1916 ~ A1408
		A1919 ~ U1406, C1407, U1495, C1496, G1517
		C1920 ~ C1496, G1517
		C1947 ~ A1483
		G1948 ~ A1418, A1483
		G1949 ~ G1419
		C1958 ~ A1418
		G1959 ~ A1418, A1483
		A1960 ~ C1484
		C1961 ~ C1484

Table S3. Interactions between h44-45 proto-SSU and mRNA.

Component	mRNA*
h44–45	* ~ #
proto-SSU #	12 ~ A1503, A1531
	13 ~ A1503, G1505, A1507
	15 ~ G1505
	16 ~ U1498
	17 ~ U1498
	18 ~ C1400 - C1403
	19 ~ A1493
	20 ~ A1492, A1493

Table S4. Interactions between the A-, P-tRNAs and the remaining components.

Component	A-tRNA #	P-tRNA #
P-DPR *	# ~ *	# ~ *
	A76 ~ A2451, C2452	C75 ~ C2063, C2064
		A76 ~ G2061, A2062, A2439, A2450, A2451
A-DPR *	# ~ *	# ~ *
	C74 ~ C2507	C75 ~ C2601, A2602
	C75 ~ C2507, C2573	A76 ~ U2585
	A76 ~ U2506, G2583, U2584, U2585, A2602	
H69–71	# ~ *	# ~ *
bridging	37 ~ A1913	11 ~ C1909
element *	38 ~ A1913	12 ~ G1907- C1909, U1923, C1924
		13 ~ C1924
		24 ~ U1923
		25 ~ G1922, U1923
h44-45	# ~ *	# ~ *
proto-SSU *	36 ~ A1493	34 ~ C1400
	37 ~ A1493	
mRNA *	# ~ *	# ~ *
	34 ~ 21	34 ~ 18
	35 ~ 20	35 ~ 17, 18
	36 ~ 19, 20	36 ~ 16, 17
	37 ~ 19	37 ~ 16

Table S5. interactions between H69-71 bridging element and the A-site (H92) of the LSU.

Component	A-site *
H69-71	* ~ #
bridging	G2549 ~ A1952, A1953
element #	G2550 ~ A1953, G1954
	C2551 ~ G1954, U1955
	U2552 ~ U1955, U1956
	U2554 ~ U1955
	C2556 ~ U1955
	G2557 ~ U1944, U1955
	C2559 ~ A1953
	A2560 ~ A1952, A1953

Table S6. Interactions between L2 residues and components of the coded proto-ribosome model.

Component	L2 *
P-DPR #	# ~ *
	C2073-4 ~ 228-9
A-DPR #	# ~ *
	A2590 ~ 238-9
	C2591 ~ 239
	U2596 ~ 243
P-site # (supp. Fig 1a)	# ~ *
	U2075 ~ 244
H69-71 bridging element #	# ~ *
	A1971 ~ 239-242

Supplementary Figures 1-3:

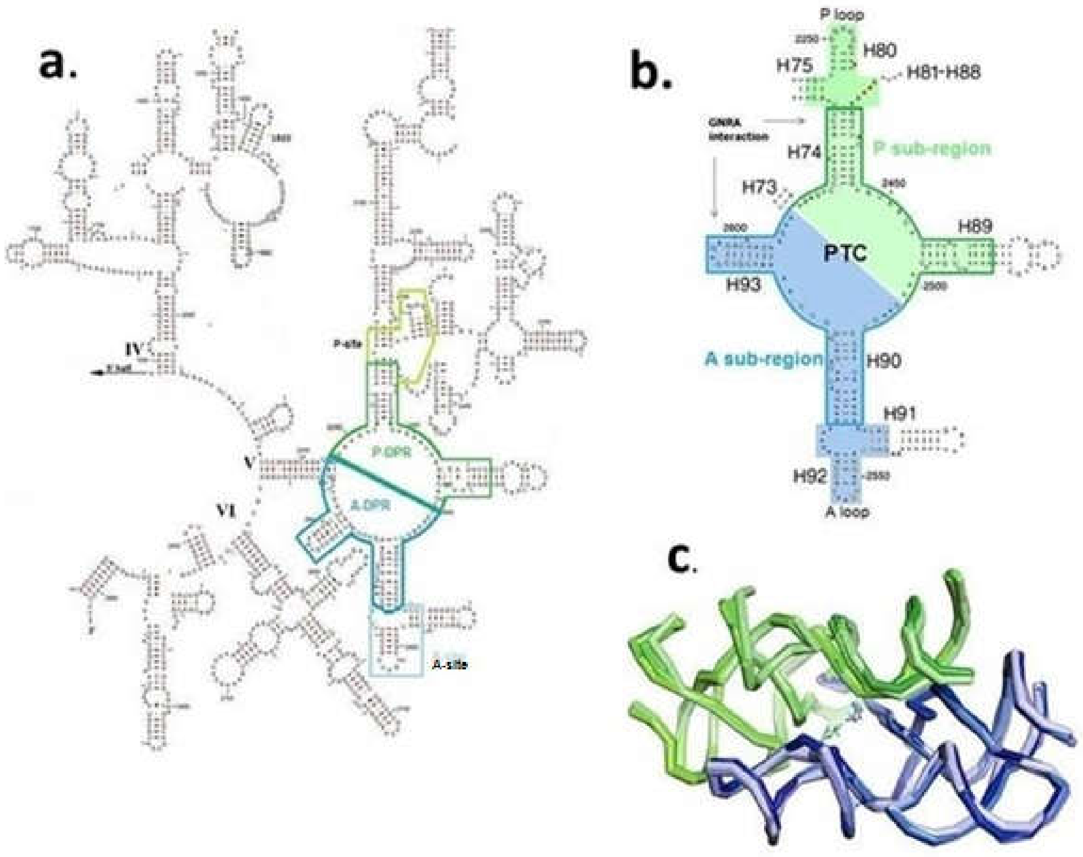

Figure S1. The symmetrical region. The symmetrical region holds 180 nucleotides related by an approximate 2-fold rotational axis. (a) Secondary scheme of the LSU 3' half with the symmetrical region drawn in a manner not portraying the symmetry. A sub-region in blue hues, P sub-region in green hues. (b) Secondary scheme of the symmetrical region drawn in a manner exhibiting the symmetry. The core of the SymR, suggested to be the remnant of the DPR (boundary marked), together with the A-, P-sites, compose the entire symmetrical region. (c) Overlap of the symmetrical region fold, with the PTC at its heart, projected along the symmetry axis, as found in the high

resolution structures of archaea (PDB code 1VQ6), three bacteria (PDB codes 2ZJR, 2AW4, 2WDL) and eukarya (PDB code 3U5D), together with the reacting amino acid analogs (PDB codes 2WDL, 1VQ6).

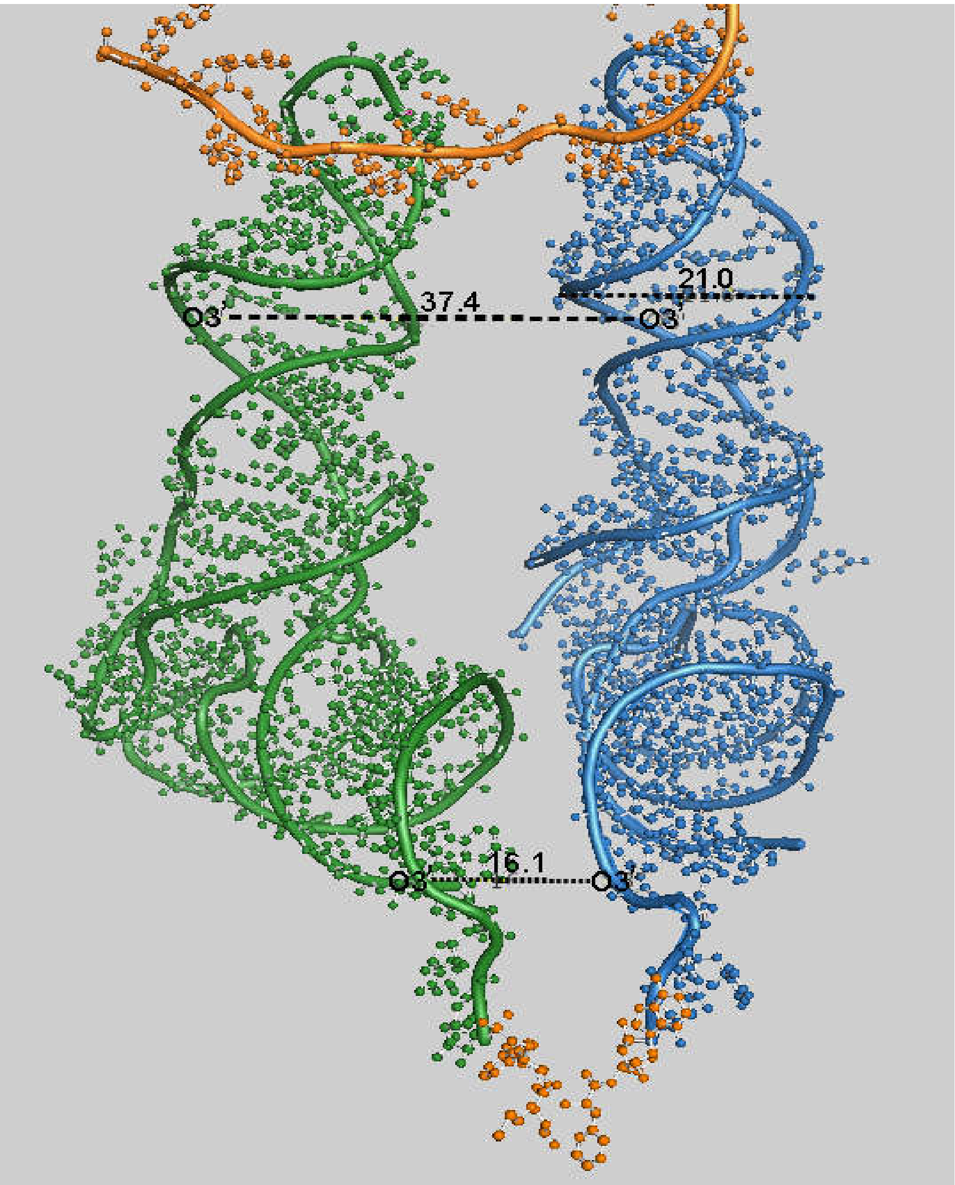

Figure S2. Geometry of two tRNAs paired to adjacent codons on mRNA**.** A-site tRNA (in blue) and P-site tRNA (in green) base paired to mRNA (in orange) as obtained from the structure of 70S ribosome (*Thermus thermophilus*, PDB code 1VY4). The amino acid reactants, at the bottom (in gold), are positioned in proximity that allows the formation of a peptide bond. The dashed lines denote distances between specific atoms. This figure is rotated by 180° around a vertical axis compared to Fig 2a in the main text.

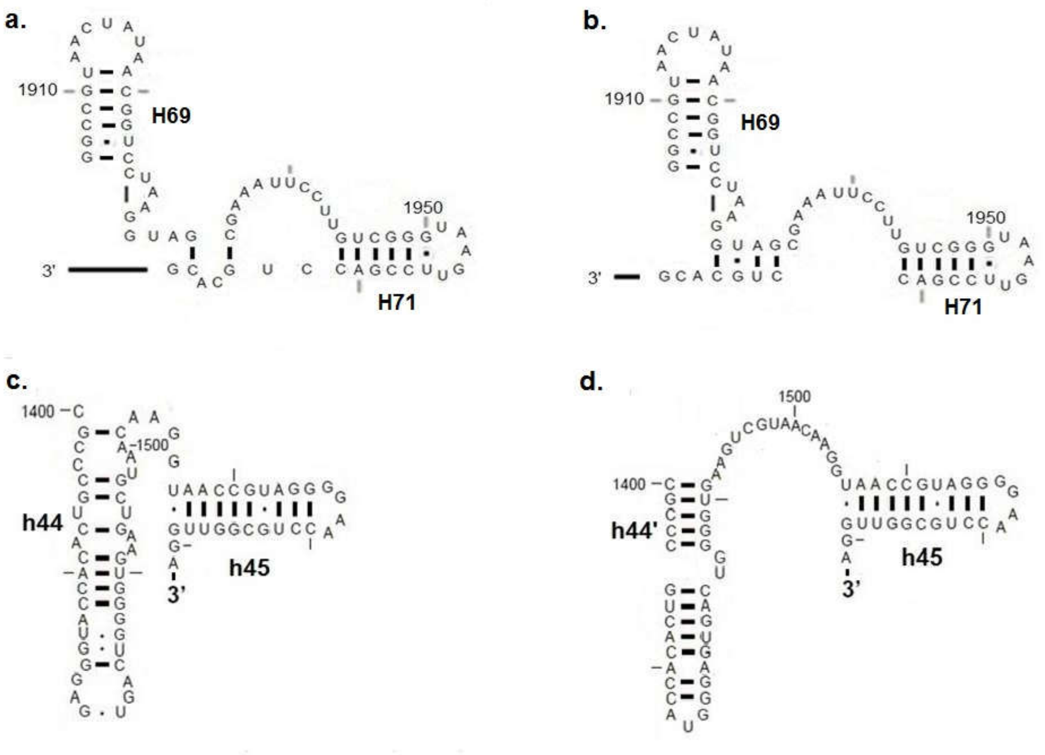

Figure S3. Ribosomal vs. predicted secondary schemes**.** (a) The secondary scheme of the H69-71 bridging element as found in the ribosome of *Thermus thermophilus* and (b) the fold prediction of its sequence. (c) The secondary scheme of the h44-45 proto-SSU element as found in the ribosome of *Thermus thermophilus* and (d) the fold prediction of its sequence.
